# Dietary choices are influenced by genotype, mating status, and sex in *Drosophila melanogaster*

**DOI:** 10.1002/ece3.4055

**Published:** 2018-05-02

**Authors:** M. Florencia Camus, Chun‐Cheng Huang, Max Reuter, Kevin Fowler

**Affiliations:** ^1^ Research Department of Genetics, Evolution and Environment University College London London UK

**Keywords:** dietary choices, *Drosophila melanogaster*, nutrition, sex differences

## Abstract

Mating causes many changes in physiology, behavior, and gene expression in a wide range of organisms. These changes are predicted to be sex specific, influenced by the divergent reproductive roles of the sexes. In female insects, mating is associated with an increase in egg production which requires high levels of nutritional input with direct consequences for the physiological needs of individual females. Consequently, females alter their nutritional acquisition in line with the physiological demands imposed by mating. Although much is known about the female mating‐induced nutritional response, far less is known about changes in males. In addition, it is unknown whether variation between genotypes translates into variation in dietary behavioral responses. Here we examine mating‐induced shifts in male and female dietary preferences across genotypes of *Drosophila melanogaster*. We find sex‐ and genotype‐specific effects on both the quantity and quality of the chosen diet. These results contribute to our understanding of sex‐specific metabolism and reveal genotypic variation that influences responses to physiological demands.

## 1 INTRODUCTION

Many species exhibit multiple changes in physiology, behavior, and gene expression following mating. Responses to mating are predicted to be sex specific, and these differences can be attributed to the divergent reproductive roles of the sexes. For example, mating induces significant changes in female insects including: elevated egg‐laying rate, increased food consumption, reduced immunity, and reduced receptivity to courting males (Chapman, Liddle, Kalb, Wolfner, & Partridge, 1995; Liu & Kubli, 2003; Rolff & Siva‐Jothy, 2002; Sgrò, Chapman, & Partridge, 1998). Less is known about the responses to mating in males, although they are predicted to primarily involve the replenishment of sperm and seminal fluid storage (Sirot, Buehner, Fiumera, & Wolfner, 2009). Several studies using *Drosophila melanogaster* have aimed to uncover the gene products that respond to mating, although these studies have examined males and females separately (Dalton et al., 2010; Ellis & Carney, 2010; Innocenti & Morrow, 2009; Lawniczak & Begun, 2004).

More recently, sex‐specific transcriptomic analysis in the seed beetle *Callosobruchus maculatus* has shed some light on gene expression affected by mating; with females showing more genes responding to mating than males (~2000 vs. ~300) (Immonen, Sayadi, Bayram, & Arnqvist, 2017). Many of the genes that responded to mating were found to be involved in metabolic pathways, suggesting sex‐specific shifts in metabolic requirements as a consequence of mating (Immonen et al., 2017). A metabolic shift can phenotypically manifest as a switch in requirement of certain macronutrients to meet the new nutritional demands of the organism. For example, several studies have examined dietary choices in flies, concluding that flies can actively make food choices according to their internal state in a sex‐specific manner (Corrales‐Carvajal, Faisal, & Ribeiro, 2016; Ribeiro & Dickson, 2010; Vargas, Luo, Yamaguchi, & Kapahi, 2010). Moreover, nutritional choices have been found to differ depending on the mating status of females. While virgin females mostly fed on carbohydrate‐based diets, mated females not only elevated their overall amount of food consumed (food quantity), but increased the concentration of protein ingested (food quality; Corrales‐Carvajal et al., 2016). In *Drosophila* females, egg production (reproductive output) has been directly linked to the nutritional state of female flies, with flies having a greater reproductive output on diets containing higher protein concentrations (Piper et al., 2014; Simmons & Bradley, 1997; Terashima & Bownes, 2004). These results allude to both a metabolic and behavioral shift for diet preference in response to a change in reproductive requirements (Kubli, 2010). However, most studies that have examined dietary responses to mating have only used one sex (females), and thus, we do not know whether males go through a similar physiological change (Corrales‐Carvajal et al., 2016; Ribeiro & Dickson, 2010; Vargas et al., 2010).

While there is evidence that the sexes differ in their dietary requirements to maximize their fitness (Jensen, McClure, Priest, & Hunt, 2015; Maklakov et al., 2008), it is currently unknown whether genetic effects on the metabolome translates into variation in behavioral responses in general, and whether the genotype of an individual will have an effect on nutritional changes induced by mating, in particular. Recently, significant genetic variation has been found in dietary requirements and dietary choices for males and female *Drosophila* (Camus, Fowler, Piper, & Reuter, 2017; Reddiex, Gosden, Bonduriansky, & Chenoweth, 2013). Together, these results suggest that genotypes vary in the rate and efficiency with which they acquire and process nutrients, and ultimately in the conversion of these nutrients into reproductive output.

Here, we ask whether males and females change dietary behavior in response to mating and if mating‐induced dietary shifts are genotype dependent. We use cytogenetic cloning techniques, and measure responses in dietary preference to mating across six genotypes in both sexes. Our results show sex‐ and genotype‐specific responses to mating. Females show a significant response in both the quantity and quality of food consumed; preferring to consume a diet with higher protein concentration following mating. In addition, we find genotype‐specific responses and link these to previously obtained fitness measurements. Males on the other hand do not show a general dietary trend following mating, although we do find complex interactions between the mating response and the genotype of the individual fly. Overall these results contribute to our understanding of sex‐specific metabolism and flexible responses to physiological requirements.

## 2 MATERIALS AND METHODS

### 2.1 Fly stock and maintenance

We used the *D. melanogaster* laboratory population LH_M_ for our experiments. This has been sustained as a large outbred population for over 400 nonoverlapping generations (Chippindale, Gibson, & Rice, 2001; Rice, 1996), maintained on a strict 14‐day regime, with constant densities at larval (~175 larvae per vial) and adult (56 vials of 16 male and 16 females) stages. All LH_M_ flies were reared at 25°C, under a 12‐hr:12‐hr light:dark regime, on cornmeal–molasses–yeast–agar food medium.

We used hemiclonal analysis to sample six haploid genomes, consisting of chromosomes X, II and III (the fourth dot chromosome is ignored), from the population. Haploid genomes are maintained without recombination and expressed in males and females. Experimental flies share a genomic haplotype (herein hemiclone), complemented by chromosomes randomly sourced from the base population (Chippindale et al., 2001). Given both target haploid genomes and complementing genomes are both sourced from the LH_M_ population, each hemiclonal fly is considered outbred. The experimental lines were not picked at random. The chosen hemiclone lines were found to have diverging levels of female productivity in a previous study, indicative of high levels of variance in female fitness in the LH_M_ population (Camus et al., 2017). We picked two lines that had overall the highest and lowest productivity (M40 and M55 respectively), whereas the other four lines were picked to represent an even spread between the two extremes (M39, M8, M91, M52).

### 2.2 Synthetic diet

We used a modified liquid version of a synthetic diet (Piper et al., 2014), prepared entirely from synthetic components (recipes are in Tables A1–A3 in Appendix S1). Previous studies have used diets based on natural components, typically live or killed yeast as the protein source and sugar as the carbon source, which do not allow precise manipulation of nutrients and are confounded by responses to other dietary components (Piper & Partridge, 2007). We synthesized two diets, protein and carbohydrate, both of which were presented to the flies during the experiment in separate capillary tubes. Each diet contained all nutritional components (vitamins, minerals, lipids) at equal concentration, with the protein diet containing amino acids and the carbohydrate diet containing sucrose. Preliminary experiments established that flies would not eat purified amino acids with the vitamin/mineral/lipid buffer, so we diluted our protein solution with 20% of a suspension of dried yeast extract, made at the same protein concentration as the synthetic solution. Given that yeast extract also contains sugars, the final protein diet then included 4% carbohydrate. We do not take into account the fractional amount of carbohydrate in our protein diet. This is mainly because yeast extract is composed of many types of carbohydrates, all of which are metabolized differently using different pathways. For this reason, we would rather have a more conservative approach and exclude carbohydrate measures. We also note that most dietary preference work in insects does not use a holidic diet, but rather yeast extract as the protein source (42% protein; Corrales‐Carvajal et al., 2016; Reddiex et al., 2013). Our diet thus provides an important step in the right direction and future work regarding the holidic medium should aim to identify the sensory cue that is missing from the protein source.

### 2.3 Dietary preference assay

Males and females were assayed separately. For each sex, the experiment was run over two identical blocks, with each block containing all experimental genotypes. Hemiclone flies from each sex and genotype were collected as virgins using CO_2_ anesthesia. Triplets of virgins were placed in individual vials containing culture medium (molasses–yeast–agar) with no added live yeast. We use triplets of flies to minimize between‐vial variance (Camus et al., 2017). Twenty vials of triplets were collected for each sex and genotype, with 10 vials allocated to the “virgin” treatment and 10 vials to the “mated” treatment. Flies in the “mated” treatment required flies of the opposite sex to mate with. For this we used, LH_M_ males and females which were reared in identical conditions to the hemiclone flies and collected as virgins in triplets. All flies were aged for 4 days in order for them to become sexually mature. Then three virgin LH_M_ flies of the opposite sex to the hemiclone were introduced to the vial with the three hemiclones of the “mated” treatment. This hextet of flies were left for 5 hours to mate, with all hemiclones subsequently being transferred to new vials containing an 0.8% agar–water mixture. Virgin flies were not perturbed during this period. We are confident that all of the focal individuals mated at least once. First, before this study we performed a trial to find the time required for mating to occur. It showed that all test flies mated within 5 hr. Second, we presented flies of the mated treatment with equal numbers of the opposite sex flies to ensure that there was no competition for mating. Finally, experimental flies were kept as virgin for a period of 4 days, which maximizes the chance of flies mating in a short period of time. Agar–water vials provide water for the flies, but have no nutritional value. Flies were kept in a controlled temperature room (25°C), 12L:12D light cycle and high relative humidity >80%.

In accordance with previous literature using this methodology (Camus et al., 2017; Reddiex et al., 2013), flies were kept in agar–water vials overnight and then supplied with two 5‐μl microcapillary tubes (ringcaps©, Hirschmann); one containing the protein solution and the other the carbohydrate solution. Capillary tubes were replaced daily, and food consumption for each fly trio was recorded for a period of 3 days. As a control, the rate of evaporation for all diet treatments was measured in six vials that contained the two solution‐bearing capillary tubes but no flies and placed randomly in the controlled temperature room. Their average evaporation per day was used to correct diet consumption for evaporation.

### 2.4 Statistical analyses

**Figure 1:**
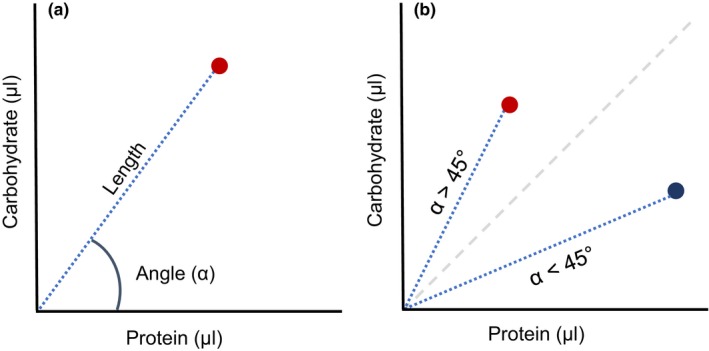
(a) Illustrative representation of food consumed in terms of quantity (length of a vector to a given data point—red dot) and quality (angle of the vector). (b) Dietary shifts represented by the angle (α) of the vector. Values of α < 45° indicate a preference for a higher concentration of protein (blue dot), α > 45° a preference for higher concentration of carbohydrate (red dot) in the diet

Given that experiments were performed separately for males and females, we analyzed separately for each sex. We used trigonometry to distinguish the quantity and quality of food consumed by flies and their changes with mating status (Figure 1). To quantify food consumed, we calculated the length of a vector starting from the origin (0,0) to the point on the carbohydrate–protein plane describing the consumption measured for a replicate trio of individuals. To quantify the quality of the food consumed, we calculated the angle (α) created between each consumption vector and the *x*‐axis. The value of the angle gives an indication of protein or carbohydrate preference. For instance, if α < 45°, then flies prefer more protein in their diets, whereas α > 45° would indicate a carbohydrate dietary preference (Figure 1).

To determine whether male and female dietary choices depend on their mating status and genotype, we subjected vector lengths and angles to a multivariate analysis of variance (MANOVA). We first removed the effect of “experimental block” by running a preliminary model, with length and angle of vector as response variables and block as a fixed effect. We then extracted the residuals of this model and used them as response variables for the main analysis. The main model had (residual) length and angle of the vectors as response variables, with mating status and genotype as fixed effects. Our two‐step model approach circumvents the fact that it is not possible to model a multivariate analysis of variance with random effects without using Bayesian statistics. Using a two‐step model approach, we were able to first remove the random effect of “block” from our data in a preliminary model and then measure the effects of “genotype” and “mating status” on the residuals of that preliminary model.

Given that we picked genotypes based on female fitness rather than sampling them randomly from the population, we included genotype as a fixed effect. We followed the MANOVA with separate univariate analyses of variance (ANOVA) for each response variable. After the univariate analyses, we performed Tukey's post hoc tests to identify genotypes that were significantly different from each other in their diet quantity and quality. All analyses were performed in R version 3.3.2 (Team, 2016).

## 3 RESULTS

**Figure 2:**
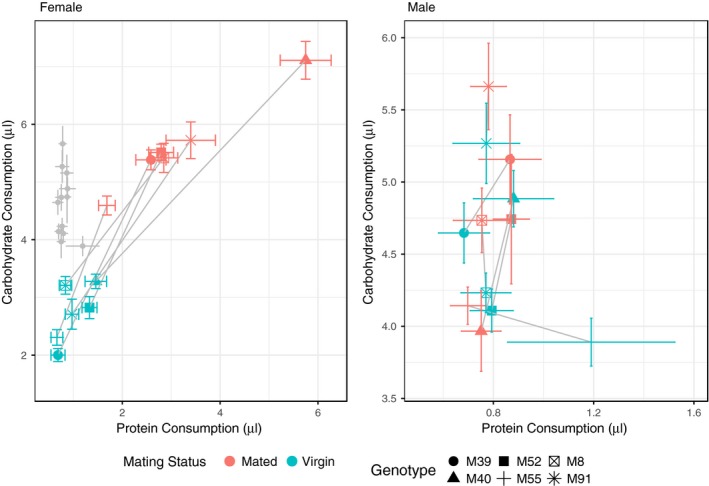
Genotype‐specific dietary response to mating for females (left) and males (right), measured as the intake (mean ± *SE*) of protein (*x*‐axis) and carbohydrate (*y*‐axis). Given the difference in scale of dietary preference between the sexes, we have also plotted the male values (gray data points) within the female plot to make the sex differences obvious

**Figure 3:**
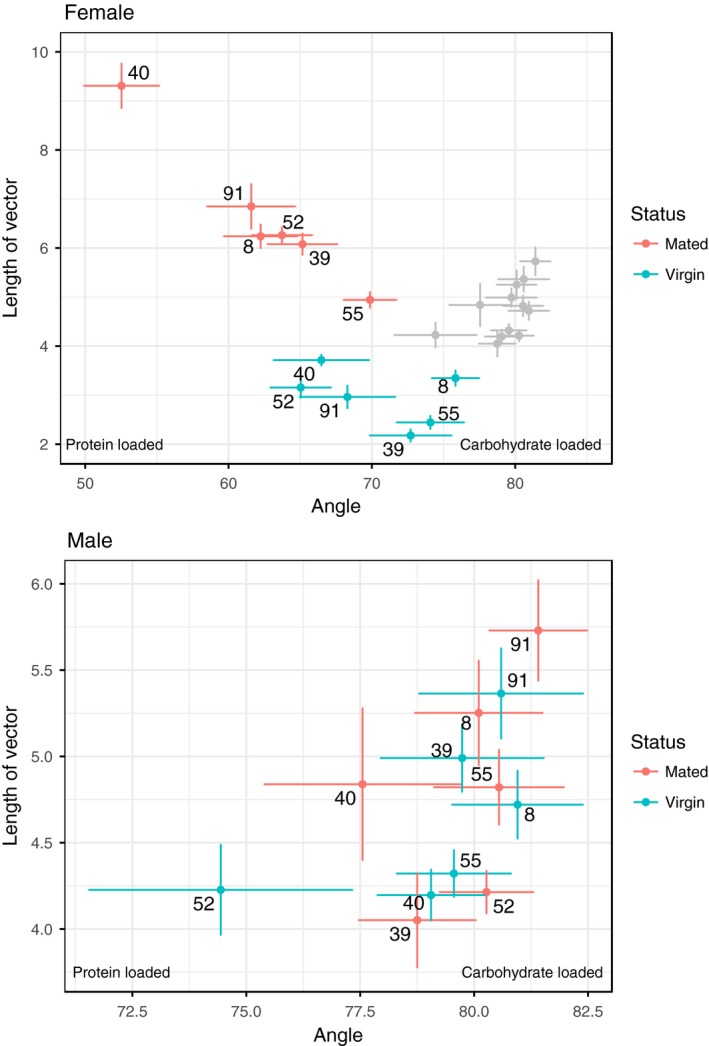
Genotype‐specific dietary responses to mating for females (top) and males (bottom) measured as the mean ± SE of angle (food quality, *x*‐axis) and length (food quantity, *y*‐axis) of the genotype‐specific vector. Given the difference in scale of dietary preference between the sexes, we have also plotted the male values (gray data points) within the female plot to make the sex differences obvious

For females, we found a significant effect of mating status on dietary choices in the overall multivariate model (Pillai's trace = 0.73657, approx. *F* = 313.160, *p* < .001, Table 1). Our data also revealed significant genetic differences on dietary choices in the overall multivariate model (Pillai's trace = 0.41642, approx. *F* = 11.833, *p* < .001, Table 1) and genetic variation for dietary shifts induced by mating (genotype × status: Pillai's trace = 0.21154, approx. *F* = 5.323, *p* < .001, Table 1). Univariate analyses revealed that across genotypes, mating increased the quantity of food consumed, and all genotypes changed their dietary quality to consume higher concentrations of protein (length: *F* = 618.8995, *p* < .001, angle: *F* = 29.4489, *p* < .001, Figures 2 and 3, Table 1; Figure A1 in Appendix S1). Furthermore, via post hoc analyses, we were able to dissect this genotypic response. For instance, genotype M40 showed the greatest response to mating by consuming more food than all the other lines and having a significantly greater protein switch than the other genotypes (Figures 2 and 3; Tables A4 and A5 in Appendix S1).

**Table 1:** Results from multivariate (MANOVA) and univariate (ANOVA) outputs for each sex

Female							Male					
MANOVA												
	*df*	Pillai	*F*	numDF	denDF	Pr(>*F*)	*df*	Pillai	*F*	numDF	denDF	Pr(>*F*)
Status	1	0.73657	313.160	2	224	<0.001	1	0.012007	1.1788	2	194	0.30984
Genotype	5	0.41642	11.833	10	450	<0.001	5	0.231438	5.1036	10	390	< 0.001
Genotype × status	5	0.21154	5.323	10	450	<0.001	5	0.117975	2.4447	10	390	0.007
Residuals	225						195					

Univariate												
Angle	*df*	Sum Sq	Mean Sq	*F*	Pr(>*F*)		*df*	Sum Sq	Mean Sq	*F*	Pr(>*F*)	
Status	1	3,739.7	3,739.7	29.4489	<0.001		1	31.7	31.740	0.8472	0.35848	
Genotype	5	3,907.7	781.5	6.1544	<0.001		5	423.3	84.665	2.2599	0.051	
Genotype × status	5	1,283.6	256.7	2.0216	<0.001		5	342.7	68.548	1.8297	0.10872	
Residuals	225	28,572.4	127.0				195	7,305.5	37.464			
Length												
Status	1	790.02	790.02	618.8995	<0.001		1	1.563	1.5628	1.7074	0.19286	
Genotype	5	184.98	37.00	28.9830	<0.001		5	44.709	8.9419	9.7690	<0.001	
Genotype × status	5	60.92	12.18	9.5448	<0.001		5	13.829	2.7658	3.0216	0.01191	
** **Residuals	225	287.21	1.28				195	178.490	0.9153			

Response variables are genotype (hemiclone line) and mating status (virgin/mated).

For males, we did not observe a significant mating response (Pillai's trace = 0.012007, approx. *F* = 1.1788, *p* = .31, Table 1) in the overall multivariate model. We did find significant differences in consumption between genotypes (Pillai's trace = 0.231438, approx. *F* = 5.1036, *p* < .001, Table 1) and mating status‐by‐genotype interaction (Pillai's trace = 0.117975, approx. *F* = 2.4447, *p* = .008, Figure 2, Table 1). Univariate analyses revealed that the significance is driven by diet consumption (length of vector) rather than dietary choices (angle of vector). While we did not find any significant effects in the univariate model where angle was a response variable, we did find genotype (*F* = 29.9.769, *p* < .001) and genotype × status (*F* = 3.0216, *p* = .012) effects on vector length.

## 4 DISCUSSION

We examined the change in dietary preference between virgin and mated flies across both sexes and six genotypes. For females, our findings echo previously obtained results (Corrales‐Carvajal et al., 2016; Ribeiro & Dickson, 2010; Vargas et al., 2010). We found a significant mating response, with mated females consuming more protein and carbohydrate than virgin females. Furthermore, there was a significant shift in the quality of the preferred diet in mated females toward increased protein concentration. We can conclude that the observed dietary switches are associated with the increased reproductive output of female flies. This is because egg production in *Drosophila* has been closely associated with increased consumption of nutrients (specifically, an increase in protein preference) in females, and our results validate these previous findings (Simmons & Bradley, 1997; Terashima & Bownes, 2004). There are many proteins involved in the process of egg production, with yolk proteins having a protagonist role in egg synthesis (Bownes, Lineruth, & Mauchline, 1991; Terashima & Bownes, 2004). For instance, disruption of yolk protein gene synthesis via gene knockouts causes severe consequences to female reproduction leading to sterility in some extreme cases (Minoo & Postlethwait, 1985). We also found significant genotype and genotype‐by‐mating effects, with both the magnitude and direction of the mating‐induced nutritional shift depending on the genotype of the fly (Table 1). For females, we know from previous data collected on these fly lines (Camus et al., 2017) that hemiclonal line M40 shows the highest levels of overall egg production, whereas M55 has the lowest fecundity of all hemiclonal lines. Our current data show that both the quantity and quality of diet are highly divergent between them (Figure 2), with line M40 consuming significantly more food than M55 (Tables A4 and A5 in Appendix S1). Line M40 also shows a larger shift in diet quality compared to M55, choosing to consume a more protein‐rich diet following mating. Although we were unable to detect any significant correlations between our current behavioral traits and previously obtained fitness data (Table A6 in Appendix S1) to support our qualitative argument, we note that our genotype sample size is low (*N* = 6). It would be interesting to further examine this relationship between nutrition and fitness by exploring whether the differences in fitness between the lines are due to nutrient sensing pathways or more efficient metabolism (or both) on a larger panel of genotypes. Nevertheless, our results suggest that metabolic pathways of the fly are tightly coupled to the nutrient sensing genes as flies actively make dietary food choices based on their genotype, mating status, and sex. Consequently, this process generates a feedback loop between reproductive needs (reproductive status) and nutrient acquisition (diet preference) in a genotype‐dependant manner.

In contrast to females, we did not observe an overall trend in the dietary response to mating for males in either quantity or quality of diet consumed (Figures 1 and 2, Table 1). One potential explanation for the lack of overall mating response is that sperm are “cheap” to make (Trivers, 1972), and so males may not need to consume much food to quickly replenish their sperm/seminal fluid storage. *Drosophila* males are capable of mating multiply and require several consecutive mating opportunities to become sperm deprived (Gromko, Gilbert & Richmond, 1984). Work on cockroaches, however, has shown that there is a nutritional cost to producing sperm, with optimal sperm production found at a protein‐to‐carbohydrate ratio of 1:2 (Bunning et al., 2015). An alternative explanation of the absence of dietary shifts in males is that as we kept experimental conditions similar for both sexes and males mated only once with a given female, then their sperm stores may have been insufficiently depleted to trigger dietary change. It would be interesting to go on to explicitly test whether sperm depleted males undergo a dietary switch similar to that in females. Although there is no general mating trend in males, we did find significant genotype and interaction between mating status and genotype effects. Closer inspection of the univariate models shows that these significant effects were driven by overall food consumption, rather than quality of the food (Table 1). These results suggest that different male genotypes chose to consume different quantities of macronutrients, and in addition respond differently to mating. The genotype‐specific mating response does not follow a general trend (unlike females), with some genotypes eating more and others eating less postmating (Figures 2 and 3). It is noteworthy that when examining the quantities of macronutrient consumption (Figure 2), much of the overall genetic variation between genotypes was in the carbohydrate axis, while protein consumption was relatively invariant between genotypes. Focal males were housed in trios, and this may have placed a premium on behavioral traits involved in coping tactics such as male–male aggression (to fend off potential rivals), or locomotory activity to decamp from rivals. Carbohydrates provide high levels of energy in a short period of time so it is plausible that carbohydrate intake could be a sensitive indicator of variation between genotypes in such coping behavior.

Our results raise questions about which evolutionary mechanisms could maintain these levels of standing genetic variance in metabolic requirement. Previous work has found genetic variance for diet preference, with significant positive between‐sex genetic correlation (Reddiex et al., 2013). Genetic variance for diet preference could be maintained due to antagonistic selection on metabolism/physiology. This is because both sexes share many metabolic traits which are under selection to optimize contrasting reproductive demands between the sexes. Alternatively, both epistatic (He, Qian, Wang, Li, & Zhang, 2010; Jakubowska & Korona, 2012; Jasnos & Korona, 2007) and/or balancing selection via temporal variation in environmental conditions (Bergland, Behrman, O'Brien, Schmidt, & Petrov, 2014) could also be mechanisms to maintain genetic variance. One study has found season‐dependent oscillating patterns of allele frequency change of central metabolic and nutrient sensing genes (Cogni et al., 2015). Those results suggest that climate factors driving latitudinal molecular variation in a metabolic pathway are related to those operating on a seasonal level within populations. Our results are in line with transcriptomic analyses performed by Immonen et al., 2017 by highlighting a mating response that is principally female specific. Immonen et al., 2017 compared the transcriptional profile of virgin and recently mated seed beetles (*C. maculatus*) in both sexes. Although both sexes showed differential expression of metabolic genes after mating, the females had 6 times more genes responding to mating than males. In the future, it would be valuable to verify whether genotype‐specific dietary preference differences in our genotypes are also linked to transcriptional responses.

Although our protein diet was composed mostly of purified amino acids plus the vitamins/minerals/lipid buffer, it contained 4% carbohydrate from yeast extract. This was necessary because our preliminary data established that flies would not eat a purely synthetic protein diet made of purified amino acids, implying that the holidic medium might lack a key sensory cue for the fly. While we cannot fully disentangle the possible effects of carbohydrate in our protein diet, that diet has a much greater protein concentration (>90%: amino acids, vitamins/minerals/lipid buffer, yeast extract) than all previous studies (~42%: yeast extract) and thus our study is more informative about the effects of protein on dietary preference.

In conclusion, we observe a dietary switch in response to mating, whereby flies changed both the quantity and quality of their preferred diet as a result of mating. Although we observed a greater response in females, both sexes showed significant levels of genotypic variance for dietary choice. These results align with published data, whereby females increase the amount of diet consumed following mating, with a general shift in the balance of macronutrients to a higher concentration of protein (Corrales‐Carvajal et al., 2016; Ribeiro & Dickson, 2010; Vargas et al., 2010). Our data suggest that there is genotypic variation in metabolic processing and/or nutrient signaling in *D. melanogaster*, ultimately causing variation in fitness. Future research ought to focus on identifying the genetic architecture of variation in the metabolic pathways that selection can act upon and examining how selection acts on dietary choices made by each sex.

## COMPETING INTERESTS

We declare no competing interests.

## AUTHORS CONTRIBUTIONS

MFC, MR, and KF conceived the study, analyzed the data, and wrote the manuscript. MFC and CCH conducted the experiment.

## DATA ACCESSIBILITY

All data can be accessed on Dryad repository (https://doi.org/10.5061/dryad.mn3gq21)
