# Supplementary material for: Dietary choices are influenced by genotype, mating status, and sex in Drosophila melanogaster
Source: Ecol Evol. 2018 May 2;8(11):5385–93. doi: 10.1002/ece3.4055 (PMC6010745; doi:10.1002/ece3.4055)
Supplement: Supplementary file 1 [file ECE3-8-5385-s001.docx]

**Table A1:** Essential and non-essential amino acid stock solutions.

| Amino acid stock solution |  | **(g/200 ml)** |  |
| --- | --- | --- | --- |
| **Essential amino acid** | | | |
| F (L-phenylalanine) |  | **3.03** |  |
| H (L-histidine |  | **2.24** |  |
| K (L-lysine) |  | **5.74** |  |
| M (L-methionine) |  | **1.12** |  |
| R (L-arginine) |  | **4.70** |  |
| T (L-threonine) |  | **4.28** |  |
| V (L-valine) |  | **4.42** |  |
| W (L-tryptophan) |  | **1.45** |  |
| **Non-essential amino acid** | | | |
| A (L-alanine) |  | **5.25** |  |
| D (L-aspartate) |  | **2.78** |  |
| G (glycine) |  | **3.58** |  |
| N (L-asparagine) |  | **2.78** |  |
| P (L-proline) |  | **1.86** |  |
| Q (L-glutamine) |  | **6.02** |  |
| S (L-serine) |  | **2.51** |  |
|  |  |  |  |
|  |  |  |  |

**Table A2:** Recipe for 200ml of protein solution

|  |  |  | **Total volume 200ml** |
| --- | --- | --- | --- |
|  | L-ile | Powder | 348mg |
|  | L-leu | Powder | 492mg |
|  | L-tyr | Powder | 252mg |
|  |  |  |  |
|  | cholesterol | 20mg/ml in EtOH | 3ml |
|  |  |  |  |
|  | CaCl2 | 1000x | 200ul |
|  | MgSO4 | 1000x | 200ul |
|  | CuSO4 | 1000x | 200ul |
|  | FeSO4 | 1000x | 200ul |
|  | MnCl2 | 1000x | 200ul |
|  | ZnSO4 | 1000x | 200ul |
|  | H_2_O |  | Up to 50ml |
|  | Total volume before autoclaving | | 50 ml |
|  |  |  |  |
|  | buffer | 10x acetate buffer base | 20ml |
|  |  |  |  |
|  | nucl/lipid soln | 125x stock | 1.6ml |
|  |  |  |  |
|  | Yaa solutions | essential amino acid stock solution (EAA) | 18.154ml |
|  |  | non essential amino acid stock solution (NEAA) | 18.154ml |
|  |  | Na glutamate solution (100mg/ml) | 5.464ml |
|  |  | Cys solution (50mg/ml) | 1.584ml |
|  |  |  |  |
|  | Vitamin stock | 47.6x stock | 4.2ml |
|  |  |  |  |
|  | folic acid stock | 1000x stock | 200ul |
|  |  |  |  |
|  | Propionic acid |  | 1.2ml |
|  |  |  |  |
|  | Nipagin | 100 g/l stock in 95% EtOH | 3ml |
|  |  | Make to total volume of 200ml with H_2_O |  |

**Table A3:** Recipe for 200ml of carbohydrate solution

|  |  |  | **Total volume 200ml** |
| --- | --- | --- | --- |
|  | sucrose | To match protein 1:1 | 6.5g |
|  |  |  |  |
|  | cholesterol | 20mg/ml in EtOH | 3ml |
|  |  |  |  |
|  | CaCl2 | 1000x | 200ul |
|  | MgSO4 | 1000x | 200ul |
|  | CuSO4 | 1000x | 200ul |
|  | FeSO4 | 1000x | 200ul |
|  | MnCl2 | 1000x | 200ul |
|  | ZnSO4 | 1000x | 200ul |
|  | H_2_O |  | Up to 50ml |
|  | Total volume before autoclaving | | 50ml |
|  |  |  |  |
|  | buffer | 10x acetate buffer base | 20ml |
|  |  |  |  |
|  | nucl/lipid soln | 125x stock | 1.6ml |
|  |  |  |  |
|  | Vitamin stock | 47.6x stock | 4.2ml |
|  |  |  |  |
|  | folic acid stock | 1000x stock | 200ul |
|  |  |  |  |
|  | Propionic acid |  | 1.2ml |
|  |  |  |  |
|  | Nipagin | 100 g/l stock in 95% EtOH | 3ml |
|  |  | Make to total volume of 200ml with H_2_O |  |

**Table A4:** Results of Tukey’s post-hoc comparisons of the quantity of food consumed by genotypes.

| **Status** |  |  |  |  |
| --- | --- | --- | --- | --- |
|  | diff | lwr | upr | p adj |
| V-M | -3.651557 | -3.940797 | -3.362317 | <0.001 |
|  |  |  |  |  |
| **Hemiclone** |  |  |  |  |
|  | diff | lwr | upr | p adj |
| 39-8 | -0.6781045 | -1.41354042 | 0.0573314 | 0.090 |
| 40-8 | 1.7149159 | 0.98409089 | 2.4457409 | <0.001 |
| 52-8 | -0.0881463 | -0.81897128 | 0.6426787 | 0.999 |
| 55-8 | -1.1030252 | -1.83385014 | -0.3722002 | <0.001 |
| 91-8 | 0.1061444 | -0.62929155 | 0.8415803 | 0.998 |
| 40-39 | 2.3930204 | 1.6621954 | 3.1238454 | <0.001 |
| 52-39 | 0.5899582 | -0.14086678 | 1.3207832 | 0.190 |
| 55-39 | -0.4249207 | -1.15574564 | 0.3059043 | 0.552 |
| 91-39 | 0.7842489 | 0.04881296 | 1.5196848 | 0.029 |
| 52-40 | -1.8030622 | -2.52924695 | -1.0768774 | <0.001 |
| 55-40 | -2.817941 | -3.54412581 | -2.0917563 | <0.001 |
| 91-40 | -1.6087715 | -2.33959649 | -0.8779465 | <0.001 |
| 55-52 | -1.0148789 | -1.74106363 | -0.2886941 | 0.001 |
| 91-52 | 0.1942907 | -0.53653431 | 0.9251156 | 0.973 |
| 91-55 | 1.2091695 | 0.47834454 | 1.9399945 | <0.001 |
|  |  |  |  |  |
| **Status x hemiclone** | |  |  |  |
|  | diff | lwr | upr | p adj |
| V:8-M:8 | -2.87928451 | -4.07475597 | -1.68381306 | <0.001 |
| M:39-M:8 | -0.16116294 | -1.37186395 | 1.04953806 | 1.000 |
| V:39-M:8 | -4.04848351 | -5.24395496 | -2.85301205 | <0.001 |
| M:40-M:8 | 3.08210852 | 1.88663706 | 4.27757997 | <0.001 |
| V:40-M:8 | -2.51175944 | -3.7072309 | -1.31628799 | <0.001 |
| M:52-M:8 | 0.03521869 | -1.16025276 | 1.23069015 | 1.000 |
| V:52-M:8 | -3.07099397 | -4.26646542 | -1.87552251 | <0.001 |
| M:55-M:8 | -1.28321346 | -2.47868492 | -0.08774201 | 0.024 |
| V:55-M:8 | -3.78231953 | -4.97779099 | -2.58684808 | <0.001 |
| M:91-M:8 | 0.62183545 | -0.57363601 | 1.8173069 | 0.858 |
| V:91-M:8 | -3.27532698 | -4.48602798 | -2.06462597 | <0.001 |
| M:39-V:8 | 2.71812157 | 1.52265012 | 3.91359303 | <0.001 |
| V:39-V:8 | -1.16919899 | -2.34924436 | 0.01084638 | 0.055 |
| M:40-V:8 | 5.96139303 | 4.78134766 | 7.1414384 | <0.001 |
| V:40-V:8 | 0.36752507 | -0.8125203 | 1.54757044 | 0.997 |
| M:52-V:8 | 2.91450321 | 1.73445784 | 4.09454858 | <0.001 |
| V:52-V:8 | -0.19170945 | -1.37175482 | 0.98833592 | 1.000 |
| M:55-V:8 | 1.59607105 | 0.41602568 | 2.77611642 | 0.001 |
| V:55-V:8 | -0.90303502 | -2.08308039 | 0.27701035 | 0.328 |
| M:91-V:8 | 3.50111996 | 2.32107459 | 4.68116533 | <0.001 |
| V:91-V:8 | -0.39604246 | -1.59151392 | 0.79942899 | 0.995 |
| V:39-M:39 | -3.88732056 | -5.08279202 | -2.69184911 | <0.001 |
| M:40-M:39 | 3.24327146 | 2.04780001 | 4.43874291 | <0.001 |
| V:40-M:39 | -2.3505965 | -3.54606795 | -1.15512504 | <0.001 |
| M:52-M:39 | 0.19638164 | -0.99908982 | 1.39185309 | 1.000 |
| V:52-M:39 | -2.90983103 | -4.10530248 | -1.71435957 | <0.001 |
| M:55-M:39 | -1.12205052 | -2.31752197 | 0.07342094 | 0.089 |
| V:55-M:39 | -3.62115659 | -4.81662804 | -2.42568513 | <0.001 |
| M:91-M:39 | 0.78299839 | -0.41247307 | 1.97846984 | 0.578 |
| V:91-M:39 | -3.11416403 | -4.32486504 | -1.90346303 | <0.001 |
| M:40-V:39 | 7.13059202 | 5.95054665 | 8.3106374 | <0.001 |
| V:40-V:39 | 1.53672407 | 0.3566787 | 2.71676944 | 0.001 |
| M:52-V:39 | 4.0837022 | 2.90365683 | 5.26374757 | <0.001 |
| V:52-V:39 | 0.97748954 | -0.20255583 | 2.15753491 | 0.216 |
| M:55-V:39 | 2.76527005 | 1.58522468 | 3.94531542 | <0.001 |
| V:55-V:39 | 0.26616398 | -0.91388139 | 1.44620935 | 1.000 |
| M:91-V:39 | 4.67031895 | 3.49027358 | 5.85036432 | <0.001 |
| V:91-V:39 | 0.77315653 | -0.42231492 | 1.96862799 | 0.598 |
| V:40-M:40 | -5.59386796 | -6.77391333 | -4.41382259 | <0.001 |
| M:52-M:40 | -3.04688982 | -4.22693519 | -1.86684445 | <0.001 |
| V:52-M:40 | -6.15310249 | -7.33314786 | -4.97305712 | <0.001 |
| M:55-M:40 | -4.36532198 | -5.54536735 | -3.18527661 | <0.001 |
| V:55-M:40 | -6.86442805 | -8.04447342 | -5.68438268 | <0.001 |
| M:91-M:40 | -2.46027307 | -3.64031844 | -1.2802277 | <0.001 |
| V:91-M:40 | -6.35743549 | -7.55290695 | -5.16196404 | <0.001 |
| M:52-V:40 | 2.54697813 | 1.36693276 | 3.72702351 | <0.001 |
| V:52-V:40 | -0.55923453 | -1.7392799 | 0.62081084 | 0.920 |
| M:55-V:40 | 1.22854598 | 0.04850061 | 2.40859135 | 0.033 |
| V:55-V:40 | -1.27056009 | -2.45060546 | -0.09051472 | 0.023 |
| M:91-V:40 | 3.13359489 | 1.95354952 | 4.31364026 | <0.001 |
| V:91-V:40 | -0.76356753 | -1.95903899 | 0.43190392 | 0.616 |
| V:52-M:52 | -3.10621266 | -4.28625803 | -1.92616729 | <0.001 |
| M:55-M:52 | -1.31843215 | -2.49847752 | -0.13838678 | 0.015 |
| V:55-M:52 | -3.81753822 | -4.9975836 | -2.63749285 | <0.001 |
| M:91-M:52 | 0.58661675 | -0.59342862 | 1.76666212 | 0.892 |
| V:91-M:52 | -3.31054567 | -4.50601712 | -2.11507421 | <0.001 |
| M:55-V:52 | 1.78778051 | 0.60773514 | 2.96782588 | <0.001 |
| V:55-V:52 | -0.71132556 | -1.89137093 | 0.46871981 | 0.699 |
| M:91-V:52 | 3.69282941 | 2.51278404 | 4.87287478 | <0.001 |
| V:91-V:52 | -0.20433301 | -1.39980446 | 0.99113845 | 1.000 |
| V:55-M:55 | -2.49910607 | -3.67915144 | -1.3190607 | <0.001 |
| M:91-M:55 | 1.90504891 | 0.72500354 | 3.08509428 | <0.001 |
| V:91-M:55 | -1.99211352 | -3.18758497 | -0.79664206 | <0.001 |
| M:91-V:55 | 4.40415498 | 3.22410961 | 5.58420035 | <0.001 |
| V:91-V:55 | 0.50699256 | -0.6884789 | 1.70246401 | 0.962 |
| V:91-M:91 | -3.89716242 | -5.09263388 | -2.70169097 | <0.001 |

**Table A5:** Results of Tukey’s post-hoc comparisons of the quality of food consumed by genotypes.

| **Status** |  |  |  |  |
| --- | --- | --- | --- | --- |
|  | diff | lwr | upr | p adj |
| V-M | 7.944674 | 5.059767 | 10.82958 | <0.001 |
|  |  |  |  |  |
| **Hemiclone** |  |  |  |  |
|  | diff | lwr | upr | p adj |
| 39-8 | -0.1837908 | -7.5190981 | 7.1515166 | 1.000 |
| 40-8 | -9.5433031 | -16.8326206 | -2.2539855 | 0.003 |
| 52-8 | -4.6656242 | -11.9549417 | 2.6236933 | 0.442 |
| 55-8 | 2.9286537 | -4.3606638 | 10.2179713 | 0.858 |
| 91-8 | -4.1572537 | -11.4925611 | 3.1780536 | 0.580 |
| 40-39 | -9.3595123 | -16.6488298 | -2.0701948 | 0.004 |
| 52-39 | -4.4818334 | -11.7711509 | 2.8074841 | 0.489 |
| 55-39 | 3.1124445 | -4.176873 | 10.401762 | 0.823 |
| 91-39 | -3.973463 | -11.3087703 | 3.3618444 | 0.628 |
| 52-40 | 4.8776789 | -2.3653568 | 12.1207145 | 0.383 |
| 55-40 | 12.4719568 | 5.2289211 | 19.7149925 | <0.001 |
| 91-40 | 5.3860493 | -1.9032682 | 12.6753668 | 0.279 |
| 55-52 | 7.5942779 | 0.3512423 | 14.8373136 | 0.034 |
| 91-52 | 0.5083705 | -6.7809471 | 7.797688 | 1.000 |
| 91-55 | -7.0859075 | -14.375225 | 0.2034101 | 0.062 |
|  |  |  |  |  |
| **Status x hemiclone** | |  |  |  |
|  | diff | lwr | upr | p adj |
| V:8-M:8 | 13.72465416 | 1.8009087 | 25.6483996 | 0.010 |
| M:39-M:8 | 2.918883072 | -9.1567633 | 14.9945295 | 1.000 |
| V:39-M:8 | 10.59332323 | -1.3304222 | 22.5170687 | 0.136 |
| M:40-M:8 | -9.578342245 | -21.5020877 | 2.3454032 | 0.257 |
| V:40-M:8 | 4.364594916 | -7.5591505 | 16.2883404 | 0.988 |
| M:52-M:8 | 1.616749139 | -10.3069963 | 13.5404946 | 1.000 |
| V:52-M:8 | 2.924861244 | -8.9988842 | 14.8486067 | 1.000 |
| M:55-M:8 | 7.758519918 | -4.1652255 | 19.6822654 | 0.588 |
| V:55-M:8 | 11.97164633 | 0.0479009 | 23.8953918 | 0.048 |
| M:91-M:8 | -0.529057978 | -12.4528034 | 11.3946875 | 1.000 |
| V:91-M:8 | 6.052456487 | -6.0231899 | 18.1281029 | 0.886 |
| M:39-V:8 | -10.80577109 | -22.7295165 | 1.1179743 | 0.117 |
| V:39-V:8 | -3.131330928 | -14.9012151 | 8.6385533 | 0.999 |
| M:40-V:8 | -23.3029964 | -35.0728806 | -11.5331122 | 0.000 |
| V:40-V:8 | -9.360059243 | -21.1299435 | 2.409825 | 0.271 |
| M:52-V:8 | -12.10790502 | -23.8777892 | -0.3380208 | 0.038 |
| V:52-V:8 | -10.79979292 | -22.5696771 | 0.9700913 | 0.107 |
| M:55-V:8 | -5.966134241 | -17.7360185 | 5.80375 | 0.878 |
| V:55-V:8 | -1.753007825 | -13.522892 | 10.0168764 | 1.000 |
| M:91-V:8 | -14.25371214 | -26.0235963 | -2.4838279 | 0.005 |
| V:91-V:8 | -7.672197672 | -19.5959431 | 4.2515478 | 0.605 |
| V:39-M:39 | 7.674440158 | -4.2493053 | 19.5981856 | 0.605 |
| M:40-M:39 | -12.49722532 | -24.4209708 | -0.5734799 | 0.031 |
| V:40-M:39 | 1.445711843 | -10.4780336 | 13.3694573 | 1.000 |
| M:52-M:39 | -1.302133934 | -13.2258794 | 10.6216115 | 1.000 |
| V:52-M:39 | 0.005978172 | -11.9177673 | 11.9297236 | 1.000 |
| M:55-M:39 | 4.839636845 | -7.0841086 | 16.7633823 | 0.973 |
| V:55-M:39 | 9.052763261 | -2.8709822 | 20.9765087 | 0.341 |
| M:91-M:39 | -3.44794105 | -15.3716865 | 8.4758044 | 0.998 |
| V:91-M:39 | 3.133573414 | -8.942073 | 15.2092198 | 0.999 |
| M:40-V:39 | -20.17166548 | -31.9415497 | -8.4017813 | 0.000 |
| V:40-V:39 | -6.228728315 | -17.9986125 | 5.5411559 | 0.844 |
| M:52-V:39 | -8.976574092 | -20.7464583 | 2.7933101 | 0.334 |
| V:52-V:39 | -7.668461986 | -19.4383462 | 4.1014222 | 0.586 |
| M:55-V:39 | -2.834803312 | -14.6046875 | 8.9350809 | 1.000 |
| V:55-V:39 | 1.378323103 | -10.3915611 | 13.1482073 | 1.000 |
| M:91-V:39 | -11.12238121 | -22.8922654 | 0.647503 | 0.084 |
| V:91-V:39 | -4.540866744 | -16.4646122 | 7.3828787 | 0.983 |
| V:40-M:40 | 13.94293716 | 2.173053 | 25.7128214 | 0.007 |
| M:52-M:40 | 11.19509138 | -0.5747928 | 22.9649756 | 0.079 |
| V:52-M:40 | 12.50320349 | 0.7333193 | 24.2730877 | 0.027 |
| M:55-M:40 | 17.33686216 | 5.566978 | 29.1067464 | <0.001 |
| V:55-M:40 | 21.54998858 | 9.7801044 | 33.3198728 | <0.001 |
| M:91-M:40 | 9.049284268 | -2.7205999 | 20.8191685 | 0.321 |
| V:91-M:40 | 15.63079873 | 3.7070533 | 27.5545442 | 0.001 |
| M:52-V:40 | -2.747845777 | -14.51773 | 9.0220384 | 1.000 |
| V:52-V:40 | -1.439733672 | -13.2096179 | 10.3301505 | 1.000 |
| M:55-V:40 | 3.393925002 | -8.3759592 | 15.1638092 | 0.998 |
| V:55-V:40 | 7.607051418 | -4.1628328 | 19.3769356 | 0.599 |
| M:91-V:40 | -4.893652894 | -16.6635371 | 6.8762313 | 0.967 |
| V:91-V:40 | 1.687861571 | -10.2358839 | 13.611607 | 1.000 |
| V:52-M:52 | 1.308112105 | -10.4617721 | 13.0779963 | 1.000 |
| M:55-M:52 | 6.141770779 | -5.6281134 | 17.911655 | 0.856 |
| V:55-M:52 | 10.3548972 | -1.414987 | 22.1247814 | 0.146 |
| M:91-M:52 | -2.145807116 | -13.9156913 | 9.6240771 | 1.000 |
| V:91-M:52 | 4.435707348 | -7.4880381 | 16.3594528 | 0.986 |
| M:55-V:52 | 4.833658674 | -6.9362255 | 16.6035429 | 0.970 |
| V:55-V:52 | 9.04678509 | -2.7230991 | 20.8166693 | 0.322 |
| M:91-V:52 | -3.453919222 | -15.2238034 | 8.315965 | 0.998 |
| V:91-V:52 | 3.127595243 | -8.7961502 | 15.0513407 | 0.999 |
| V:55-M:55 | 4.213126416 | -7.5567578 | 15.9830106 | 0.990 |
| M:91-M:55 | -8.287577896 | -20.0574621 | 3.4823063 | 0.462 |
| V:91-M:55 | -1.706063431 | -13.6298089 | 10.217682 | 1.000 |
| M:91-V:55 | -12.50070431 | -24.2705885 | -0.7308201 | 0.027 |
| V:91-V:55 | -5.919189847 | -17.8429353 | 6.0045556 | 0.892 |
| V:91-M:91 | 6.581514465 | -5.342231 | 18.5052599 | 0.804 |

**Table A6:** Correlation coefficients between female angle/length and female fitness.

|  | angle (mated) | length (mated) | angle (virgin) | length (virgin) |
| --- | --- | --- | --- | --- |
| fitness | -0.62 | 0.67 | -0.15 | 0.19 |
| *p-value* | *0.185* | *0.149* | *0.770* | *0.715* |

**Figure A1:** Dietary shift between virgin and mated females (top) and males (bottom), denoted as P:C ratio of dietary intake of virgin (x-axes) and mated (y-axes) flies, where grey line is the line of equality. Values below the grey line indicate a carbohydrate nutritional shift, and values above the line a protein preference shift, from virgin to mated flies.
